# No time for that now! Qualitative changes in manuscript peer review during the Covid-19 pandemic

**DOI:** 10.1093/reseval/rvaa037

**Published:** 2021-01-05

**Authors:** Serge P J M Horbach

**Affiliations:** 1 Department of Political Sciences, Danish Centre for Studies in Research and Research Policy, Aarhus University, Bartholins Allé 7, Aarhus C, 8000, Denmark; 2 Faculty of Social Sciences, Centre for Science and Technology Studies (CWTS), Faculty of Social Sciences, Leiden University, Wassenaarseweg 62A, AL Leiden 2333, The Netherlands

**Keywords:** scholarly publishing, Covid-19, publication process, peer review, quality criteria

## Abstract

The global Covid-19 pandemic has had a considerable impact on the scientific enterprise, including scholarly publication and peer-review practices. Several studies have assessed these impacts, showing among others that medical journals have strongly accelerated their review processes for Covid-19-related content. This has raised questions and concerns regarding the quality of the review process and the standards to which manuscripts are held for publication. To address these questions, this study sets out to assess qualitative differences in review reports and editorial decision letters for Covid-19 related, articles not related to Covid-19 published during the 2020 pandemic, and articles published before the pandemic. It employs the open peer-review model at the *British Medical Journal* and *eLife* to study the content of review reports, editorial decisions, author responses, and open reader comments. It finds no clear differences between the review processes of articles not related to Covid-19 published during or before the pandemic. However, it does find notable diversity between Covid-19 and non-Covid-19-related articles, including fewer requests for additional experiments, more cooperative comments, and different suggestions to address too strong claims. In general, the findings suggest that both reviewers and journal editors implicitly and explicitly use different quality criteria to assess Covid-19-related manuscripts, hence transforming science’s main evaluation mechanism for their underlying studies and potentially affecting their public dissemination.

## 1. Introduction

The 2020 global Covid-19 pandemic has brought about major changes to several aspects of society, including science and scientific publishing (Andersen et al. 2020; Bian and Lin 2020). Witnessing a strong increase in the number of submitted manuscripts to both pre-print servers and traditional journal outlets (Colavizza et al. 2020), researchers have been at their marks to fight the pandemic with novel scientific results. In addition, many journals and publishers have adapted swiftly to facilitate the quick dissemination of novel findings (Putman, Ruderman and Niforatos 2020). Several journals installed *fast-track* systems for Covid-19-related content, making sure the review process was executed as fast as possible and avoiding unnecessary publication delay. In fact, several studies demonstrated these efforts to be highly effective, with average time between submission and publication being reduced by nearly 50% (Horbach 2020; Putman et al. 2020). While laudable, this acceleration raised a host of questions about the content and quality of the review process: are Covid-19-related papers equally scrutinized and hold against the same quality standards as other manuscripts?

Concerns about the speed and quality of the peer-review process at scholarly journals are all but a recent development. For decades, scholars have been complaining about the system being slow (Tosi 2009; Nguyen et al. 2015), conservative (Luukkonen 2012), inconsistent (Peters and Ceci 1982), prone to bias and error (Smith 2006; Tennant et al. 2019), and incapable of distinguishing high from low-quality academic work (Schroter et al. 2008). Several scandals and hoaxes showing how apparently easy non-sensible, problematic, or fraudulent research may slip through the peer-review system has put the system under further pressure (Bohannon 2013). Nevertheless, manuscript peer review still holds a core position in the scholarly publishing landscape and as such is a main evaluative mechanism in research. Indeed, many still maintain their faith in the current system, regarding it as the best available option to safeguard the quality of academic work and uphold the integrity of the academic literature.

However, new and even louder concerns have been raised during the current pandemic, among others after the quick spread of news about several high-impact studies, in well-reputed journals, which had to be retracted following evidence of research misconduct or the unavailability of underlying data (Marcus and Oransky 2020; UNESCO 2020). Examples include studies in the *Lancet* and the *New England Journal of Medicine*, claiming the (in)effectiveness of certain drugs to cure—or reduce negative effects of—Covid-19.

In this article, we will build on earlier work regarding the peer-review process in times of the global pandemic (Horbach 2020). Rather than assessing merely the speed of the process, we will now examine qualitative changes in the actual content of the review process. In order to do so, we take advantage of the open peer-review model installed at various medical journals, in which documentation regarding the review process, including review reports, editorial decision letters, and author responses to both, are made publicly available. In particular, we study the review process for articles in two medical journals, using in-depth qualitative analysis of the review documents to examine differences between Covid and non-Covid-related manuscripts and between manuscripts reviewed during and before the pandemic. Herewith, we aim to shed light on how the pandemic has affected the scholarly publication system and its core mechanism of quality control and research evaluation.

## 2. Theoretical background

Manuscript peer review serves many purposes, several of which have been in flux over the past decades with new expectations of the system appearing. Traditionally, the system of journal peer review has been expected to serve primarily as a gatekeeping mechanism, filtering fit from unfit manuscripts for the limited space available in journals’ print copies, as well as a mechanism to improve the quality of a manuscript based on reviewers’ feedback (Csiszar 2016; Moxham and Fyfe 2017; Tennant and Ross-Hellauer 2020). Throughout its history, several other expectations have been added to this list, including the detection of fraudulent material; the provision of fair and equal chances to publish for all authors, irrespective of their background and personal characteristics; and the establishment of a hierarchy of published results, usually coupled to journal status and reputation (Baldwin 2018; Horbach and Halffman 2018; Pontille and Torny 2015).

Tied to these changing expectations, the scholarly community has witnessed a diversification of review models. By now, a host of different ways of performing manuscript peer review is installed at different journals and publishers (Horbach and Halffman 2019; Halffman and Horbach 2020). Most notably, these models include diversity in the level of anonymity of both authors and reviewers (Pontille and Torny 2014), leading to systems ranging from double- or even triple-blind review to radically open peer-review models (Ross-Hellauer 2017). In a quest to enhance the transparency of the system, to make reviewers accountable for their reports and to give credit to those performing reviews, increasingly many journals are currently adopting systems of open review, either publishing review reports, reviewers’ identities or both (Smith 1999; Bravo et al. 2019; Horbach and Halffman 2020). Others have however expressed concerns about these new models increasing publication delay or decreasing the willingness to review, particularly among junior scholars (van Rooyen, Delamothe and Evans 2010; Ross-Hellauer, Deppe and Schmidt 2017).

Studies of peer review, both as a formal process or system and as a daily scholarly practice, have a long tradition (Zuckerman and Merton 1971; Cole, Cole and Simon 1981; Peters and Ceci 1982). Scholars from Science and Technology Studies, have for decades been occupied in how credit is distributed true publishing and review practices, as well as examining consistency across journals and reviewers (Peters and Ceci 1982; Biagioli 2002). More recently, these endeavours have also included studies on the economic and commercial aspects of the publishing industry, including specific interest in novel business models created by open access schemes (Mirowski 2018; Fyfe et al. 2020), and the role of digital technologies in transforming publishing and review practices (Bohlin 2004; Walker and Rocha da Silva 2015).

While some evidence is gathered on the procedural level, documenting review procedures and formal regulations, little study has gone into the actual practice of review, nor into the content of individual review reports. Part of this has certainly been due to a lack of data availability; with review commonly being an opaque practice, performed by individual researchers and not shared with the wider public or research community. This has changed due to the establishment of open peer-review models and increasingly many journals adhering to systems in which review reports are publicly shared. Such systems now warrant studies of peer review’s content that were previously particularly challenging to perform. In addition, the collaboration between researchers and academic publishers has allowed some to peak into the review process of certain journals (e.g. Bravo et al. 2019; Squazzoni et al. 2020).

Taking advantage of these open review formats, some studies have aimed to classify reviewer comments, using quantitative and qualitative methods to distinguish between various types of content in review reports (Herber et al. 2020). The majority of these studies, however, used quantitative methods to assess for instance the ‘quality’ of review reports (e.g. Landkroon et al. 2006; Henly and Dougherty 2009) or they assessed review instructions or review guidelines rather than actual review reports (Davis et al. 2018; Malički et al. 2019). These studies usually demonstrate differences in review depending on specific context, for instance, differences between research fields, academic publishers, or manuscript types. However, they therewith stay on a rather procedural level, not assessing differences between actual review content or actors’ decisions based on this content.

Two notable exceptions to this are the recent studies by Herber et al. (2020) and Siler and Strang (2017). In the former, the authors use a focused mapping review and synthesis to compile a list of no less than 77 different elements found within the content of a review report, including request for additional clarification, suggestions for references, and requests for details or nuances to a paper. The latter study uses a combination of open review reports and a survey among journal authors to examine the criticisms and subsequent changes that arise in the course of peer review. They find that papers challenging theoretical perspectives obtain higher levels of criticism and change, particularly regarding methodology, while those that offer a new perspective or that extend or combine established perspectives were less criticized and changed (Siler and Strang 2017).

## 3. Methods

To assess the process and content of manuscript peer review at academic journals, and the potential changes it faced during the 2020 Covid-pandemic, we used the open peer-review model at two medical/life science journals. We sampled articles from the *British Medical Journal* (*BMJ*) and the online journal *eLife*, for three distinct categories. Both journals constitute prominent journals in their field, with the *BMJ* having a long tradition in publishing in Medicine, and *eLife* being a relatively young life science journal. While *BMJ* was one of the first journals to install a formal system using external reviewers at the end of the 19th century (Baldwin 2017; Fyfe et al. 2017), both journals are known for being particularly innovative regarding their review procedures, among others testified by their early adoption of open review models in which review reports are openly shared with the research community alongside published articles (Horbach and Halffman 2018, 2020). This reflects their interest in and endeavours to optimize their peer-review systems, making them particularly suitable journals to use in the present study. The date of sampling was 23rd April 2020. Articles were sampled in the following three categories:

- Covid-19-related content published after the start of the pandemic. Decisions about what articles were related to Covid-19 were based on the classification of the journal itself, in case of BMJ, and inclusion in the CORD19 database, in case of eLife (CWTS 2020). On the day of sampling, the most recently published articles were sampled. In the remainder of this text, this category will be referred to as the Covid.
- Articles published after the start of the pandemic (i.e. after January 2020) not related to Covid-19. Again, relatedness to Covid-19 was based on the classification of the journal itself, in case of *BMJ*, and inclusion in the CORD19 database, in case of eLife. Again, the most recently published articles were sampled on the sampling date. In the remainder of this text, this category will be referred to as the non-Covid.
- Articles published before the Covid-19 pandemic emerged. For this category, we sampled the most recently published articles as of exactly 1 year before the sampling date, that is, the most recently published articles in *BMJ* and *eLife* before 23rd April 2019. In the remainder of this text, this category will be referred to as the pre-Covid.

For all categories, five articles per journal were included in the sample, that is, 30 articles in total. Because only four articles were published on Covid-19 in BMJ at the sampling date, the fifth article was added to this category once it was published on 5th May. Only articles classified as ‘Research articles’ by the respective journals were eligible for inclusion in the sample. The classification of Covid-19 relatedness of the articles was based on the *BMJ*’s and CORD19’s classification, rather than assessment by the authors, to increase transparency and replicability of the sampling strategy.

For all sampled articles, we subsequently gathered all available information and documentation regarding the review process. This particularly included: bibliographic information of the articles, the articles’ dates of submission and publication, the review reports, editorial decision letters based on these review reports, author responses to review reports, the number of peer reviewers involved, the number of review rounds a paper went through, the average length of review reports, and whether articles had gone through previous rounds of review at other journals. The latter information was retrieved from the editors’ decision letters. For the *BMJ*, we also gathered the commentaries published as readers’ responses to the articles. These commentaries were sampled on 29th April 2020 (and at 12th May for the article added on 5th May to the sample of Covid-papers). It should be noted that *eLife* does not publish the full review reports but merely summaries of the most essential comments and feedback.

An overview of the sampled articles including some basic information on their review process can be found in Supplementary material S1. In total, the sampled articles have received 148 individual review reports, counting the number of reviewers involved per paper and the number of rounds of reviews. The total stock of analysed material amounts to nearly one hundred documents (treating the set of reviews from all reviewers per review round as a single document), covering over 750 pages.

Upon collecting the material, all material was read and manually coded using an inductive coding scheme loosely inspired by the classification of Herber et al. (2020). This coding scheme was chosen as a basis because of it being the most recent such coding scheme (to the knowledge of the authors) and its comprehensibility. Because Herber et al.’s scheme was specifically developed to code review reports of qualitative studies, several codes were added related to other study methods and data types, including codes referring to statistics and quantitative data. Supplementary file SB contains an overview of the coding scheme, listing all codes that occurred at least three times including a short description of each code. In particular, reviewer comments were coded, based on their content (i.e. the aspect of the manuscript they deal with) and the sort of remark made (e.g. whether the comment is a request, a suggestion, a praise, a criticism, etc.). The open reader responses published alongside articles in the *BMJ* were coded in the same way, as were the editorial decision letters based on review reports. Following up on this coding, the materials and their codes were read through yet another time to fine-tune coding and the increase consistency across coding. Subsequently, comparisons between article categories were conducted and apparent differences across categories were further scrutinized. No dedicated coding software was used. As this manuscript is primarily interested in the qualitative differences in peer-review processes brought about by the global pandemic, we refrain from quantitative analyses of our codes. In fact, sample sizes are too small to infer any meaningful statements from such statistical analyses, with characteristics of individual reviewers or manuscripts likely accounting for a substantial share of any variation found.

## 4. Results

### 4.1. Some numbers: quicker but no less elaborate reports

We start off this section with some brief numerical characteristics of the review process. The number of reviewers per paper is largely consistent between the Covid and the other categories, with on average 3.3 reviewers per paper for the Covid sample and 3.45 reviewers per paper for the other two categories. Some further characteristics regarding the duration of the review process as well as the average length of review reports are provided in Table 1. First, consistent with earlier findings about the acceleration of the review process for Covid-19-related manuscripts, Table 1 shows that articles in the Covid-category went through review much faster than articles in the other categories. This can partly be explained due to the fact that all manuscripts in the Covid-category were accepted after a single round of peer review, whereas manuscripts in the other two categories went through 1.6 rounds of review on average.

**Table 1:** Some numerical characteristics of the review process for articles in the three sample categories

	Covid	Non-Covid	Pre-Covid
Average duration of review process (in number of days between submission and publication)	83.8	199.7	201.7
Average length of review reports (in number of words)	2779.1	3015.2	3262.2
Average article length (in number of pages)	14.2	20.2	23.4
Average length of review reports relative to article length (in number of words per article page)	264.0	126.0	142.6

Even though review reports were hence delivered much faster, they, on average, seem not to be less elaborate. The length of review reports, in number of words, is fairly stable across categories. In fact, as manuscripts in the Covid-category tend to be shorter than those in other categories and longer manuscripts may be expected to yield longer review reports, the average length of reports per article page is even much higher for the Covid-category than for the other categories.

### 4.2. Qualitative changes in review reports

In this section, we will dive deeper into the qualitative content of the review reports. In particular, four aspects showing remarkable differences between the categories will be highlighted: (1) requests for additional experiments, (2) suggestions for alternative manuscript structures, (3) demands to tone down conclusions, and (4) the general nature of comments made. First and foremost however, we note that the general structure of review reports is largely stable across the three sample categories (Covid, non-Covid, and pre-Covid). As was noticed before, amongst others by Herber et al. (2020), reviewers—at least on accepted manuscripts—almost always start off their review indicating general praise for the manuscript, followed by explicit praise for aspects or sections that they found particularly valuable, before they put forward any negative feedback. The same holds true for the reviews among our samples. Another consistency across the three categories was the emergence of several codes as being (most) prevalent, including the comments on textual or linguistic aspects, and requests for additional clarification, justification of methods, or discussion of results. Despite these general consistencies among review reports’ structure and code prevalence, several noticeable differences between reports from the three distinct categories emerged. However, almost all noticeable differences occurred between the Covid-category when compared to the other two categories. Very little systematic variation was found between the non- and pre-Covid samples. Hence, any diversity reported, is unlikely to be attributable to general changes due to the pandemic, but relates specifically to the Covid-19 content. In this section, we will discuss these differences, providing illustrative examples from review reports in the relevant samples.

#### 4.2.1. Requesting new experiments

The most striking difference between review reports in the Covid-category compared to the others concerns the nature and prevalence of requests for additional experiments or the collection of novel data. In both the non-Covid and pre-Covid categories, a quite common comment in review reports would highlight the fact that data or experiments in the current manuscript are insufficient to properly answer the review question. Consequently, reviewers would point out the need for further data collection or the performance of wider (control) experiments. A typical example of such a comment pointing to the need for additional control experiments is:

‘Regarding selectivity of MtTMEM175, the authors arrive at a mechanistic conclusion that differs completely from the earlier conclusion by the Jiang group, but they do not test the effect on selectivity of mutating the residue (L35) proposed to play a key role in the earlier study. Selectivity of the L35A mutant should be studied and the results included in the interpretation’. (*eLife*, non-Covid-category)

In contrast, for articles in the Covid-category, these requests, although they exist, are less common and tend to be of a different nature. Many of the reviews in the Covid-category simply do not contain comments related to further data or wide experimentation. Moreover, in the cases where such comments are present, they tend to be much more conditional, acknowledging the fact that data collection might be difficult under pandemic conditions and inviting the authors to include data or experiments only if this can be done ‘easily’ or ‘quickly’. This tendency becomes amongst other instances apparent in:

‘We would like you to address this concern in greater depth, and consider what if any additional measures could be (quickly) incorporated to improve confidence in the medical history (e.g., online records from other hospitals).’ (*BMJ, Covid-category*)

In a nutshell, gathering clinical data during an outbreak of a new pathogen is of high importance. The manuscript should benefit from a revision before publication. If data are available at the time of the revision and if the authors chose to review their paper, the paper could be updated with new available data on the outcome of the patients in their cohort. (*BMJ, Covid-category*)

In some cases, the editors or reviewers even specifically admit that they lower their standards due to the importance of communicating novel findings that might be relevant in the pandemic. This is for instance the case, when *eLife’*s editors accept a paper that updates a previous model established by the authors with parameters matching the Covid-19 pandemic:

Our conclusion is that it does expand on your previous work, perhaps not to the extent that we usually like to see in such papers. However, in light of the importance of the topic vis-vis the ongoing COVID-19 epidemic, we intend to retain your paper for publication in *eLife.* (*eLife, Covid-category*)

Another way in which comments relating to the need of additional data or experiments were differently expressed in the Covid-category compared to the other two categories, was through a larger share of comments asking for additional discussion, toning-down of conclusions, or acknowledging limitations. We will return to this topic in Section 4.2.3.

Instead of requesting for additional data or experiments, reviewers and editors seemed to be just as likely to ask for clarification of existing methodology and data collection. For all three categories, such requests were among the most prevalent comments in review reports and editorial decision letters. These requests also include the more elaborate reporting for (statistical) variables and collected data. Illustrative examples include:

the main limitation of this manuscript is the limited information that is provided on the medication used in the different patient groups. There was a very high usage of steroids and antiviral drugs, which both could have dramatic effects on viral dynamics and clinical outcomes. The authors do mention this in their limitation section, but in my view, they need to include more data on the different drugs used in their results section. (*BMJ, Covid-category*)

The authors only gave brief description of different characteristics. Statistical analysis should be performed with statistics calculated and p values listed in each table. (*BMJ, Covid-category*)

Although the whole-cell currents of MtTMEM175A recorded from HEK293 cells appear large compared to mock-transfected cells and a point mutation (T38A) has changed ion selectivity of the channel, it would still be interesting to know whether the authors have ever confirmed expression of the protein on a biochemical level*.* (*eLife, non-Covid-category*)

#### 4.2.2. Suggesting alternative structures or formats

A second notable difference between review reports and editorial decision letters in the Covid-category as compared to the other two categories, concerns comments about the manuscripts’ structure or overall configuration. These comments commonly take the form of:

We believe that more efforts are necessary to communicate the results and interpretations to the general reader who is less of an expert in the details of the specific technology and methodologies used here. A substantial revision of the manuscript structure and style is required. (*eLife*, non-Covid-category)

In general, suggestions or critiques requiring major revisions to the manuscript’s structure are less prevalent in the Covid-category. In case they are mentioned, they are more guided in the form of concrete suggestions rather than mere criticisms pointing out the issue of poor structure or organization. The following examples are particularly illustrative of this, with the former being a comment from a pre-Covid review report and the latter from Covid review report:

While the study includes an impressive volume of detailed, quantitative analyses, the manuscript suffers from poor organization and lack of structure, which makes it challenging for the reader to tease apart the real significance of the authors' contributions. (*eLife*, pre-Covid-category)

You present a large number of laboratory parametres [sic] in your text. Given the large volume, I would encourage you to report only on the parametres [sic] that are significantly different between the two groups or that are clinically surprising, and refer your reader to the table that captures the rest. (*BMJ*, *Covid-category*)

In one instance, a particularly interesting suggestion for an alternative manuscript organization was given in the Covid-category. Due to a lack of data and the likelihood of more reliable data appearing in due course, the reviewers, followed by the editors, suggest to turn the manuscript into a ‘Living document’ accruing updates when novel data become available:

He [one of the statistical reviewers] and others also wondered whether this should also be a ‘Living Review’ - you would need to include some methodology about this, e.g. when you would update. (*BMJ*, *Covid-category*)

#### 4.2.3. Toning down conclusion

As was already noted in Section 4.2.1., requests for additional data or experiments were less prevalent and tended to be of a different nature in reviews in the Covid-category. However, this does not mean that reviewers do not point out that the current data or analyses fail to provide a sufficient basis for answering the research question or underpinning current conclusions. Rather than solving this through requests of additional data, these issues were more commonly addressed through a request for further discussion of the findings, the acknowledgement of limitations, or the weakening of conclusions. In the following example from a review report in the *BMJ* Covid-category, both instances come together, with reviewers first asking for more analyses and, if this is not possible, asking for further discussion instead:

The timing and types of drugs used must affect the interpretation of viral load and shedding times. Severe patients were more likely to be on steroids and have higher viral load; which came first? If you can't describe and analyse this in more detail then you should discuss this more. (*BMJ*, *Covid-category*)

The following quote from an *eLife* Covid review report shows an example in which reviewers would typically demand additional (control) experiments in the non- or pre-Covid categories. However, in this case of a Covid paper, the reviewer suggests the topic to be ‘clearly addressed in the Discussion’:

There are several pieces of data in the manuscript that indicate that EsxA may not be the only factor, or that there may be additional receptors for EsxA on M cells. For example, […]. This possibility should be clearly addressed in the Discussion. (*eLife, Covid-category*)

The following example takes this even a step further. Here, the poor quality of available data is actually suggested to be the manuscript’s main conclusion rather than a point of critique or reason to reject the manuscript:

We also note the low quality of most of the studies in the review. Many editors were concerned that the studies may not translate well to other settings. Perhaps that is one of the important conclusions of this review, and you should be more forceful in urging scepticism [sic] and caution. (*BMJ*, *Covid-category*)

We need to emphasize that suggestions to damper conclusions or to acknowledge study limitations are surely not unique to the *Covid-category*. In fact, they frequently appear in the other categories as well and even seem to make up a considerable portion of the essential comments and requested revisions. However, what does seem to be specific to the Covid-category, is the suggestion to use this as a stylistic or linguistic technique to address issues of data unavailability or the infeasibility of coming to stronger conclusions under pandemic circumstances.

#### 4.2.4. Nature of comments: suggestions, questions, requests, or critique

Apart from differences in the content referred to in reviewers’ comments, we also analysed the nature of the comments made, thereby distinguishing between suggestions, questions, requests, criticism, or praise.

Given the speed at which reviews were performed in the Covid-category, one might expect reviewers to be more likely to state comments that require less engagement from the reviewer. For example, one might expect comments relating to a lack of theoretical underpinning of an argument to be accompanied by a simple request to add more references, rather than the suggestion for specific literature to be included. However, we did not find evidence for this. On the contrary, it seems that reviewers are at least as engaged with Covid-manuscripts’ content as with other manuscripts. As was pointed out already in Section 4.2.2, reviewers of Covid-category manuscripts were more likely to accompany their requests for changes in manuscript structure by concrete suggestions of how to do so. The same holds true for comments related to additional references and comments related to writing style or linguistic concerns regarding the article. The following example is from a review report on a non-Covid-manuscript:

In general, the manuscript suffers unclear and complex wording; the introduction is inflated, providing many dissociated ideas and concepts without a clear narrative. The results section is overly long and detailed, while some analyses are missing and some could be moved to supplementary section (for the benefit of experts). (*eLife, non-Covid*-category)

Such comments, merely critiquing the current manuscript without giving suggestions on how to improve it, were very uncommon among the Covid-category reviews. Interestingly, no clear differences can be witnessed between the pre- and non-Covid categories. Any effects on Covid-related content can hence arguably not be attributed to general shifts in reviewers’ attitudes or levels of cooperation that might have emerged due to the pandemic.

### 4.3. Post-publication comments from general readership

The open reader responses to published articles in *BMJ* provide a further lens on how (post-publication) review processes might have changed during the Covid pandemic. A first, and by far most prominent difference, can be found in the number of such responses posted. At the moment of data collection, the articles in the Covid-category yielded an order of magnitude more responses since their publication (ranging from a week to two months before data collection) than the articles in the pre-Covid-category (being published more than a year ago). In comparison, the non-Covid category received hardly any responses at the time of data collection, with only two out of five articles receiving at least one commentary. Also, responses tended to be much more elaborate in the Covid category, commenting on a wide range of issues, rather than pointing out single concerns or potential mistakes. Even though reader comments were coded in the same way as review reports and editorial decision letters, we will refrain from presenting any quantitative data on this coding, due to the small number of reader commentaries available, especially for the non-Covid category. Instead, the coding will be used to inform a general discussion of some overall patterns in the post-publication response letters.

A particularly prominent feature of reader responses to Covid-19-related articles was the connection they tend to make to the broader literature. In their responses, readers commonly linked a study’s findings to other research, potentially published after the study being commented on. By doing so, they actively discuss the study findings, provide alternative interpretations, or point to a study’s wider relevance. Other frequently occurring comments in readers’ responses include requests for further clarification or suggestions for future research building on the current study. As such, reader responses for this category tend to be rather neutral in their tone. A last prevalent type of comment among responses to Covid-19 papers comprises more general discussions or thoughts on the pandemic, apparently only loosely inspired by the manuscript responded to.

In contrast, responses to articles in the pre- and non-Covid categories are strongly characterized by a much more critical tone, with readers pointing out potential flaws in the methodology or (statistical) analyses, or highlighting inconsistency either internally in the manuscript or compared to other study’s findings. Herewith, reader responses mainly take the role of an extension of the review process, whereas responses to articles in the Covid-category commonly take a role in extending the discussion within the article itself.

## 5. Conclusion

This article has aimed to shed light on shifting practices of manuscript peer review under the global pandemic caused by Covid-19. Previous studies have indicated review processes in medical journals for Covid-19-related content have been considerably faster than review processes before the pandemic, or for content not related to Covid-19 (Barakat et al. 2020; Horbach 2020). This raised the obvious question of whether reviews were performed equally thoroughly and whether any qualitative changes occurred within reviewers’ comments or editorial decisions. To examine these questions, this article used the open peer-review model of two medical journals to assess whether differences between review practices could be observed between Covid-19-related articles, articles not related to Covid-19 published during the 2020 pandemic, and articles published before the pandemic.

The results of our analyses suggest that, even though Covid-19-related content was reviewed substantially faster, review reports do not seem to be less thorough, judging by their length and the nature of comments made by reviewers. In fact, reviewers seem to be at least as engaged with the manuscripts’ content, providing detailed comments and suggestions for improvement, rather than merely pointing out flaws or gaps.

Nevertheless, several important differences in the content of the review reports and editorial decision letters were observed. Most notably, Covid-19-related content attracted fewer requests for additional experiments or data gathering, less requests for major structural revisions of manuscripts, and more suggestions to address potential shortcomings in alternative ways. The latter may include suggestions to simply acknowledge limitations or rephrase conclusions, to only add additional data if this does not require substantial (time) investments, or even to state the lack of high-quality data as one of the study’s main conclusions. Some of these observed changes in review content constitute plausible explanations for the earlier findings that Covid-19-related content passes the peer-review process faster than other manuscripts.

This suggests that reviewers and editors use different criteria for Covid-19-related papers compared to other manuscripts submitted to their journals. Both actors seem to be somewhat milder regarding Covid-19-related manuscripts, accepting shortcomings that would usually not pass an editorial evaluation or the journal’s selection mechanism. In several cases, reviewers and editors specifically refer to the Covid-19 pandemic and the difficulties it raises for data collection or the necessity it creates to disseminate novel findings to account for their decisions. However, most changes were not explicitly attributed to the pandemic, suggesting that actors either did not feel the need to stress this explicitly or that shifts in quality standards occurred unconsciously. Similar to comments in reviewer reports and editorial decision letters, the open reader responses to the articles in *BMJ* tended to be milder for articles in the Covid-category compared to pre- and non-Covid-articles. This was reflected in their generally more neutral tone, connecting studies to the wider literature, rather than questioning or criticizing methodology or analyses. Alternatively, instead of characterizing the observed differences as deviations from ideal review practices, one could argue that the differences indicate the ability of the review process to adequately respond to changes in context and demand. It arguably indicates an agility and adaptability of a system that is usually typified as conservative, traditional, or even ‘out-dated’.

We stress however that these are all merely general patterns to which several exceptions exist. Indeed, requests for additional data were also found in the Covid-category, suggestions to damper conclusions or acknowledge additional limitations were surely not exclusive to this category, and readers have posed critical comments to Covid-manuscripts just as they have done to others. Nevertheless, all patterns described here were prominent across the sampled materials and are hence likely to be indicative of wider patterns in pandemic review practices.

## 6. Discussion

### 6.1. Limitations

This study may have suffered from various limitations. First, even though it analyses a substantial amount of documents, these documents were related to a relatively small set of manuscripts being published in just two different journals. Individual characteristics of either the manuscripts, the reviewers involved, or the journal (including their editors and their editorial process), might have impacted the study findings. Caution should hence be exercised when generalizing the current findings to other contexts and the wider Covid-19 literature.

Second, the data used for all analyses in this study were collected in the relatively early phases of the pandemic. This might have impacted on the analyses, for example, because Covid-19 articles that had to go through a lengthy review process could not be included in the study sample. In addition, it is unclear whether the differences found in our study indicate a lasting effect or are rather signs of a temporary shift in review attitudes. Nevertheless, the differences reported here seem to be important, especially because knowledge uptake from novel scientific articles—particularly those published in well-reputed journals such as *BMJ* and *eLife—*has been extremely fast during the pandemic. Indeed, both policy and clinical decisions had to be made fast. Therefore, content published in the early phases of the pandemic is particularly likely to have had policy and clinical implications.

Third, our study relies on the analyses of accepted manuscripts only. Due to the unavailability of relevant documents for manuscripts that were rejected by journals, we were not able to include these manuscripts in our study. Similar concerns have been raised in the past and constitute a major hurdle in research on academic peer-review practices (Squazzoni et al. 2020). In particular, this limitation makes us unaware of whether differences found generalize to all manuscripts or whether they are specific to only accepted manuscripts. This implies us to exercise caution when we conclude that journal editors and reviewers are milder towards Covid-19-related content or might be willing to more easily accept those manuscripts to their journals.

### 6.2. Impact on science, policy, and society

In evaluating scholarly manuscripts dealing with content related to Covid-19, journal editors, peer reviewers, and readers alike seem to be somewhat milder in their opinions, using different quality criteria and being satisfied with potentially lower standards. In particular, they seem to acknowledge that gathering additional data, performing additional experiments, or extensively restructuring a manuscript requires considerable efforts. With researchers, policymakers, and society at large direly looking for additional knowledge about the virus and ways how to fight it effectively, ‘There is no time for that now’.

Lowering the bar on the number of (control) experiments conducted or data sets included can have multiple implications for the quality of the science conducted. For several decades now, multiple scholars have claimed science to be in a ‘reproducibility crisis’ (Guttinger 2020; Nelson et al. 2020). While an abundance of potential causes of this has been proposed (e.g. Bird 2018; Camerer et al. 2018; Flis 2019), small sample sizes and a lack of study power have been among the most frequently mentioned (e.g. Ioannidis 2005; Wicherts 2017; Bird 2018). Hence, the tendency to refrain from requests to gather additional data might contribute to the wider spread of the replication crisis, or the failure of early results translating into effective clinical interventions.

Interestingly, changes in the review process and editorial evaluation seem to concern not merely a loosening of standards but also involve a shift in quality criteria used. For example, the case of the updated version of a clinical model presented in Section 4.2.1 reflects a shift from the importance of novelty towards a focus on clinical or societal relevance. Therewith, the Covid-19 pandemic seems to have, at least temporarily, influenced fundamental characteristics of research evaluation and impacted traditional notions of what is perceived as ‘high quality’ research (Mårtensson et al. 2016; Langfeldt et al. 2019).

As mentioned before, a lack of clear differences between the pre- and non-Covid categories, suggests that any changes regarding review practices for Covid-papers, are not likely to be attributable to general cooperative attitudes that might have emerged during or due to the global pandemic (Derrick 2020). Instead, though this is slightly speculative, it seems that reviewers that agreed to perform reviews of Covid-papers, and perform them relatively quickly, engage in their task of improving manuscript quality rather than acting as strict gatekeepers. This is most clearly reflected in the nature of their comments being more cooperative, more commonly including specific suggestions rather than mere points of critique or open questions and requests.

As a final remark, we note the observation that changes in the review and editorial practices seem to occur across a range of different actors involved. Particularly, peer reviewers seem to be at the heart of most changes, initiating decisions about whether to make specific requests or give suggestions for manuscript improvement. Subsequently, journal editors commonly follow suit, but they seem to take a less active role in the changes described. Lastly, as indicated by *BMJ*’s open reader responses, also the wider community of readers and researchers in their post-publication commentaries to papers, seem to comment in qualitatively different ways to Covid-19-related content. Hence, the Covid-19 pandemic seems to have influenced the peer-review process across its full range of stakeholders. It will be interesting and important for future research to keep monitoring these changes, to assess whether they have a lasting effect on science’s central mechanism of research evaluation.

## Supplementary data

Supplementary data are available at *Research Evaluation Journal* online.

## Supplementary Material

rvaa037_Supplementary_Data
