# Supplementary material for: No time for that now! Qualitative changes in manuscript peer review during the Covid-19 pandemic
Source: Res Eval. 2021 Jan 5:rvaa037. doi: 10.1093/reseval/rvaa037 (PMC7928627; doi:10.1093/reseval/rvaa037)
Supplement: rvaa037_Supplementary_Data [file rvaa037_supplementary_data.zip › Supplementary material B - Coding scheme.docx]

| Code | Description |
| --- | --- |
| Accusation of misconduct / QRP | *Accusations of potentially problematic research practices, concerning either misconduct or QRPs* |
| Acknowledge limitation | *Request or demand to acknowledge additional study limitations* |
| Add information / analysis of subgroups | *Requests or suggestions to analyse a particular subgroup of the study sample separately and report the results of this analysis.* |
| Additional discussion requested | *Requests or demands for additonial discussion of the study findings* |
| Additional explanation / clarification | *Request for additional explanation or clarification of specific statements or aspects of the manuscript or the study* |
| Additional justification / motivation for methods | *Requests or demands for a justification of the methods used.* |
| Additional recommendations requested | *Requests, demands or suggestions to add additional recommendations based on the study findings* |
| Alternative presentation suggested | *Suggestion or request for alternative presentation of specific elements of the manuscript, e.g. suggestions on how to clarify a figure or requests to alter the study narrative to increase readability.* |
| Comments on reference / inclusion of additional references | *Comments related to the references used, e.g. suggestions or requests for additional references, or corrections to current references* |
| Downsize conclusion | *Request, suggestion, demand, or recommendation to tone down or nuance the conclusions* |
| Eliminate statistical analysis | *A suggestion or request to remove one of the (statistical) analyses from the manuscript* |
| Highlighting inconsistency / flaws in data sampling / methods | *Comment pointing out inconsistency or flaws in the collection of data or other methodological aspects concerning data sampling or analysis* |
| More interpretation of data | *Requests, suggestions or recommendations to add more interpretation of the study data, e.g. discussing potential causes or consequences of observed patterns* |
| New analyses | *Request, suggestion, demand, or recommendation for new analyses, i.e. involving already existing data.* |
| *New experiments* | *Request, suggestion, demand, or recommendation for new experiments, i.e. involving the collection of new data.* |
| Other way of presenting results | *Requests or suggestions for alternative presentation of the study results, e.g. using figures rather than tables, or suggestions for additional variables to be added to tables* |
| Pointing out flaw in argument | *A comment pointing at a flawed argument* |
| Pointing out misalignment of conclusions with results | *A comment pointing at inconsistency between the study results and conclusions* |
| Protocol / case report should be added | *Requests or demands to add additional documentation concerning experiments, such as study protocols or case reports.* |
| Style / structure of manuscript | *Comments about the style or structure of the manuscript, e.g. suggestion to move parts to another section* |
| Textual comments | *Comments about linguistic elements, e.g. spelling mistakes, incorrect grammar, or unclear writing* |

**Supplementary material B - Coding Scheme**

List of codes and a brief description of their content, for all codes used at least three times in coding the review reports, editorial decision letters and open reader responses. Codes are sorted alphabetically.
